# Failing upwards: Genetics-based strategies to improve antibiotic discovery and efficacy in *Mycobacterium tuberculosis*

**DOI:** 10.3389/fcimb.2022.932556

**Published:** 2022-09-15

**Authors:** Francesca G. Tomasi, Eric J. Rubin

**Affiliations:** Department of Immunology and Infectious Diseases, Harvard T. H. Chan School of Public Health, Boston, MA, United States

**Keywords:** antibiotic resistance, tuberculosis, bacterial genetics, chemical genetic profiling, CRISPRi, TnSeq, drug discovery

## Abstract

Therapeutic advances in the 20th century significantly reduced tuberculosis (TB) mortality. Nonetheless, TB still poses a massive global health challenge with significant annual morbidity and mortality that has been amplified during the COVID-19 pandemic. Unlike most common bacterial infectious diseases, successful TB treatment requires months-long regimens, which complicates the ability to treat all cases quickly and effectively. Improving TB chemotherapy by reducing treatment duration and optimizing combinations of drugs is an important step to reducing relapse. In this review, we outline the limitations of current multidrug regimens against TB and have reviewed the genetic tools available to improve the identification of drug targets. The rational design of regimens that sterilize diverse phenotypic subpopulations will maximize bacterial killing while minimizing both treatment duration and infection relapse. Importantly, the TB field currently has all the necessary genetic and analytical tools to screen for and prioritize drug targets *in vitro* based on the vulnerability of essential and non-essential genes in the *Mtb* genome and to translate these findings in *in vivo* models. Combining genetic methods with chemical screens offers a formidable strategy to redefine the preclinical design of TB therapy by identifying powerful new targets altogether, as well as targets that lend new efficacy to existing drugs.

## Graphical Abstract

Existing chemical and genetic tools can be used to improve antibiotic discovery against tuberculosis. Left: High-throughput screening of chemical libraries derived from natural products (such as soil and fungal extracts), as well as from synthetical small-molecule collections, can identify anti-tubercular compounds for further development into clinically available drugs. Right: Genetic tools including transposon sequencing (TnSeq; top), proteolytic degradation (bottom left), and CRISPR interference (CRISPRi; bottom right) can be used to identify and characterize genes whose products are required in relevant laboratory growth conditions (such as macrophage models) for target discovery and prioritization. Importantly, chemical and genetic tools can also be combined to validate drug targets, identify synergistic activities between existing molecules and hypothetical drug targets, and gain valuable insights while designing combination antibiotic therapies. Figure created on BioRender.com 
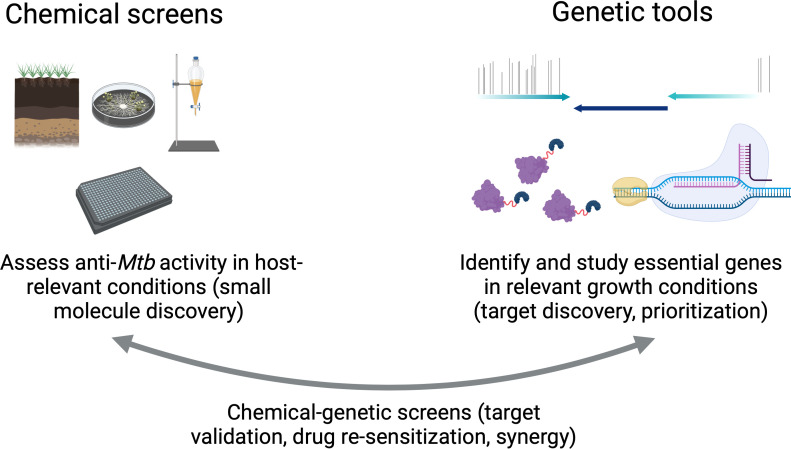

## Current challenges in tuberculosis therapy

A persistent disease requires a persistent response. The 2021 Global Tuberculosis Report published by the World Health Organization (WHO) estimates that 1.5 million people died of TB in 2020 and that recent progress in reducing TB incidence has stalled globally during the COVID-19 pandemic (W.H.O, 2021). The social, political, and economic challenges of TB control are exacerbated by the months-long regimens currently required to treat *Mycobacterium tuberculosis* (*Mtb*). Strengthening research programs to improve TB therapy will synergize with global health efforts to increase access to, as well as success of, treatment.

The first anti-tuberculous drug, streptomycin, was discovered by Selman Waksman, Elizabeth Bugie, and Albert Schatz in 1943. Patients who received streptomycin saw initial clinical improvement but eventually developed resistance; single-drug treatment did not significantly improve TB mortality in the long term (Gernez et al., 1948; Regniers et al., 1949; Tempel, 1949). These discoveries ushered in an era of small-molecule discovery against *Mtb* and the development of a multidrug therapy to minimize rates of acquired genetic resistance (Connolly et al., 2007). This multidrug therapy is administered for long periods of time (4–9 months with drug susceptible TB) to circumvent *Mtb*’s ability to develop phenotypic resistance, in which a subpopulation of metabolically altered cells tolerates antibiotic exposure and requires a longer time to eradicate (Connolly et al., 2007; Hicks et al., 2018). Longer treatment times also cause more frequent and serious side effects ranging from malaise to neuropathy. Adverse events during long-course antibiotic treatment have been major barriers to eliminating TB worldwide. When a drug regimen is taken fully as prescribed, relapse is still estimated to occur in about 5% of patients with drug-susceptible after 6 months of first-line treatment and in about 20% of patients after 4 months (Colangeli et al., 2018). *Mtb* is also intrinsically resistant to most of the antibiotics used to treat other bacterial infections, which requires TB-specific drug discovery programs and reduced overlap with other antibiotic discovery programs.

The emergence and spread of multidrug-resistant (MDR) TB pose an additional and massive medical and economic burden. MDR TB is defined by *Mtb* that is resistant to at least one first-line TB drug in addition to isoniazid or rifampicin (W.H.O, 2021). Extensively drug-resistant (XDR) TB is resistant to multiple first-line TB drugs and is even more complicated to treat. Recent advances in MDR and XDR TB therapy have significantly improved treatment outcomes and reflect ongoing progress in anti-tubercular drug discovery, including the discovery and implementation of pretomanid, bedaquiline, and linezolid (Conradie et al., 2020).

Long-term relapse-based experiments in mice established shorter and more effective regimens by combining pretomanid and bedaquiline with linezolid or moxifloxacin and pyrazinamide (Tasneen et al., 2011; Li et al., 2017; Xu et al., 2019). The Nix-TB trial on humans (ClinicalTrials.gov #NCT02333799) found that combining bedaquiline, pretomanid, and linezolid could treat patients with highly drug-resistant TB in 6 months (compared to traditional 20-month regimens), although not without significant adverse events (Conradie et al., 2020). The shift to an all-oral, more effective, and shorter regimen for drug-resistant TB is a major success. Nonetheless, its duration and side effects warrant continued research, as access to simpler therapies remains a priority for drug susceptible, MDR, and XDR TB. Recent clinical trials have also seen promise in shorting treatment times for drug-susceptible TB (ClinicalTrials.gov #NCT02410772): a 4-month rifapentine-based regimen including moxifloxacin was determined to be non-inferior to the current standard 6-month regimen (Dorman et al., 2021). How can we continue to drive down treatment times?

In this review, we examine the tools available to identify new antibiotic targets in whole cells, to discover compound mechanisms of action, to search for off-target effects, and to use existing drugs in new ways. Identifying new antibiotic targets can be accomplished through high-throughput small-molecule screens against whole cells as well as target-based discovery using genetic tools. We can also apply these strategies to lend efficacy to existing drugs and quantify combinatorial effects between different experimental treatments. Historically, maximizing synergy between antibiotic targets was not a priority when establishing TB regimens. Here, we encourage a synergy-focused framework for designing new treatment courses that focus on well-tolerated combination antibiotics that maximize bacterial killing. This strategy is uniquely poised uniquely poised to improve both antibiotic discovery and efficacy of candidate treatments.

## Identifying new antibiotic targets

### Chemical screens

Systematic screens of soil, bacterial, or fungal extracts as well as synthetic chemical libraries have seen the most translational success so far in antibiotic discovery (Tommasi et al., 2015). A benefit to a chemical screen approach is the convenience of starting with small molecules with promising physiochemical properties that can be further modified. A major barrier to TB drug development is cell wall penetration, and chemical screens select for compounds that can already enter *Mtb* cells. Many first- and second-line TB drugs are derived from natural products including rifampicin and aminoglycosides such as streptomycin (Mdluli et al., 2015). These approaches work by testing compound libraries for their ability to block bacterial growth *in vitro*, usually under standardized aerobic growth conditions, and has enabled the identification of particularly accessible bacterial targets and pathways that are susceptible to existing compounds.

While historically effective, chemical library screening *in vitro* using standard laboratory growth conditions has now led to recurring targets (such as MmpL3) and redundancies in hits, with similar small molecules repeatedly being discovered in different libraries. In fact, the most-used antibiotics across bacterial infections block the same small subset of targets (Payne et al., 2007). This is also a reflection of library screen design: the same approaches to identifying compounds will yield the same results. In the era of antibiotic resistance, diversity of mechanism is crucial to bypass existing resistance mechanisms. Indeed, more recent chemical screens have been conducted in host-relevant growth conditions including low pH, different carbon sources, nitric oxide stress, hypoxia, and granuloma assays (Cho et al., 2007; Huang et al., 2018; Early et al., 2019).

Pyrazinamide is a breakthrough TB drug that underscores the value of testing multiple growth conditions and mimicking the host environment. On its own in a standard broth microdilution assay, pyrazinamide does not have appreciable growth-inhibiting effects on *Mtb*. However, when added under hypoxic conditions, pyrazinamide becomes highly sterilizing and has been critical to reducing TB treatment times from multiple years to several months (Wade and Zhang, 2004). This example shows the importance of evolving chemical screens to identify critical compounds that may not otherwise be uncovered using standard laboratory growth conditions.

Despite substantial efforts in systematic chemical library screens, treatment of drug-susceptible TB has remained largely unchanged since the mid-20th century, highlighting the need for creative new approaches. One such approach involves re-sensitization studies, in which compounds are screened for inhibition of stress responses instead of direct killing of bacterial cells. In one study, a small molecule was identified that inhibited *Mtb* tolerance to oxidative stress, acid stress, and isoniazid by inhibiting respiration (Flentie et al., 2019). In this case, use of this molecule with current TB treatment regimens could potentially reverse isoniazid resistance.

Library screens using specialized strains and other growth conditions described above such as macrophage screens, hypoxic media, or caseum-like environments are likely to improve translation from *in vitro* hits to *in vivo* success [reviewed in (Dartois and Barry, 2013)]. In 2009, Christophe et al., published a cell-based assay that used confocal fluorescence microscopy to screen for compounds that blocked *Mtb* replication within macrophages. Through this screen, they identified dinitrobenzamide derivatives with potent anti-tubercular activity and showed that these compounds inhibited the essential cell wall synthesis enzyme DprE1 (Christophe et al., 2009). At the same time, a study was published on the synthesis of benzothiazinones with anti-TB activity that used reverse genetics reveal that DprE1 is also the target of this compound (Makarov et al., 2009). Since these findings, DprE1 inhibitors with different types of chemical scaffolds have been developed and are being investigated as potential TB therapeutics [reviewed in (Chikhale et al., 2018)]. In subsequent sections of this review, we will highlight advances in maximizing the utility of compounds identified in chemical screens.

### Genetic tools

Starting with a small-molecule and identifying the target for promising hits is the empirical, top-down approach to drug discovery. Recent progress in bacterial genetics, high-throughput sequencing, and medicinal chemistry such as fragment-based drug discovery (Scott et al., 2009) offers promise to dedicating increased efforts to the bottom-up approach of target-based drug development. In this strategy, a single-gene product or mechanism is identified as an effective drug target based on biological studies. Inhibition of this target is shown *in vitro* to be sufficient to confer a meaningful therapeutic effect. The identification of new targets and mechanisms of bacterial killing will also likely circumvent pre-existing resistance mechanisms and thus be applicable to drug susceptible, MDR, and XDR TB.

In the past, functional deletions such as genetic knockouts have been used to validate hits but with two major drawbacks. First, targets that are essential for bacterial viability cannot be knocked out; second, antibiotics do not always fully inhibit their targets. Instead, genetic knockdowns—titrating levels of gene expression or gene products—offer a more realistic simulation for potential inhibitors of essential and non-essential genes and offer insight on the “vulnerability” of a target: how much (or how little) target inhibition is required for a therapeutic effect. Three primary genetic approaches have been used in TB research to study individual targets and their vulnerability: transposon sequencing (TnSeq), proteolytic degradation systems, and CRISPR interference (CRISPRi).

### TnSeq

TnSeq works by mapping random transposon integration sites in saturated mutant libraries: the ability or inability of a locus to sustain transposon insertion is indicative of that gene’s requirement for growth in the condition tested. The predecessor to TnSeq, transposon site hybridization (TraSH), first identified the complete set of *Mtb* genes required for growth under different conditions. This tool combined high-density insertional mutagenesis using phage containing a *mariner*-based transposon with microarrays to map pools of mutants (Sassetti et al., 2001). It has since been replaced with TnSeq, which uses next-generation sequencing to quantify marked transposon insertions across the genome (Zhang et al., 2012; van Opijnen and Camilli, 2013; DeJesus et al., 2017).

In addition to providing insight on gene essentiality, TnSeq has been a powerful tool to identify drug targets, mechanisms of action, or antibiotic resistance and to characterize so-called “conditional essentiality”. Although some genes may not be required for growth in standard laboratory media, they may no longer sustain transposon insertions in other conditions. Recent work has shed light on genes required for host-relevant conditions such as cholesterol catabolism (Griffin et al., 2011) and different genetic backgrounds *in vivo* using collaborative cross mouse panels (Smith et al., 2022). TnSeq has also been used to investigate phenotypic variation in clinical *Mtb* strains: certain genes are differentially required across clinical strains, including *katG*, which activate the pro-drug isoniazid, a first-line TB drug (Carey et al., 2018). This finding shows the ability for TnSeq to predict resistance mechanisms to antibiotics: a loss-of-function mutation in *katG* would confer isoniazid resistance by failing to activate this drug in cells.

Studies on the conditional essentiality of *Mtb* genes across strains and growth conditions have produced extensive amounts of data on the “druggable” genome in *Mtb*: genes that could be further explored as putative drug targets because they are indispensable for growth or confer increased susceptibility to antibiotics when inactivated (discussed below). Following up on hits of interest and transforming findings into phenotypic assays for both chemical screening and medicinal chemistry can help establish more productive strategies for TB drug discovery.

### Genetic knockdowns

Proteolytic degradation systems and CRISPRi allow us to study essential genes by providing titratable ways to reduce their abundance in bacterial cells. Proteolytic degradation works by tagging a gene of interest with a degradation signal: inducible expression of an accessory molecule then shuttles tagged proteins to endogenous proteases (Kim et al., 2011). Different expression levels of this accessory molecule allow for a range of protein knockdown. CRISPRi instead uses targeted transcriptional repression. An inducible nuclease deactivated Cas9 (dCas9) is guided to a target gene by a single-guide RNA (sgRNA) molecule with complementarity to the target DNA sequence (Rock et al., 2017). The sgRNA molecule also contains a short protospacer-adjacent motif (PAM) for initial dCas9 recognition, which disrupts the target DNA and facilitates binding of sgRNA to its complement (Rock et al., 2017). This interaction physically blocks RNA polymerase from being able to initiate transcription or fully elongate mRNA. Different PAM sequences confer a range of dCas9 binding, which, in turn, modulates the level of knockdown (Rock et al., 2017).

Both tools facilitate target-based drug discovery by allowing researchers to define the vulnerability of specific genes or pathways. Chemicals can also be screened against hypomorph libraries to analyze potential off-target effects and to validate mechanisms of action. For instance, proteolytic degradation systems were recently used in a large chemical-genetic screen to combine small molecules with a pool of *Mtb* strains depleted in essential genes (Johnson et al., 2020). Because these hypomorphs are hypersensitive, this approach was found to yield more hit compounds of interest than whole-cell chemical screens alone, and their effects on different strains shed light on compound mechanisms of action. More than 40 new compounds were found to target previously established *Mtb* targets including DNA gyrase, cell wall biosynthesis, and RNA polymerase. An inhibitor of a new target was also identified, which interfered with the essential *Mtb* efflux pump EfpA. This inhibitor, which was effective against an EfpA hypomorph, was then chemically modified for increased potency against a wild-type *Mtb* strain (Johnson et al., 2020). This example shows the utility of combining genetic knockdowns with whole-cell chemical screens to identify small molecules that would not be unearthed by exclusively screening wild-type cells.

Recently, a large-scale CRISPRi screen quantified the vulnerability of essential genes and pathways to predict *Mtb*’s susceptibility to an antibiotic targeting that gene or pathway (Bosch et al., 2021). A considerable challenge in target-based drug discovery is the development of a selective drug from scratch with suitable pharmacokinetic and toxicological profiles. Identifying highly vulnerable targets that require only small levels of inhibition for a clinically relevant phenotype would maximize the likelihood of success, because lower levels of target engagement would need to be achieved for compound efficacy. Advances in medicinal chemistry such as fragment-based drug design and dynamic combinatorial chemistry have the potential to further increase the likelihood of success for compound development (Scott et al., 2009) [reviewed in (Ladame, 2008)].

Multiple highly vulnerable targets have been identified that do not yet have inhibitors against them. Several promising pathways have emerged from recent work that were even more vulnerable than the targets of current TB antibiotics, such as protein folding and secretion, metabolism, DNA replication, cell division, and tRNA synthetases (Bosch et al., 2021). The latter group—aminoacyl tRNA synthetases (aaRS)—was found to be highly vulnerable regardless of the *Mtb* strain tested. Because aaRS have conserved active sites, compounds designed to target them could target multiple synthetases and reduce rates of antibiotic resistance (Kovalenko et al., 2019). Efforts are already underway to develop aaRS inhibitors against *Mtb* (Gudzera et al., 2016; Li et al., 2017; Soto et al., 2018; Kovalenko et al., 2019) [reviewed in (Kim et al., 2020)]. These enzymes are already the targets of the anti-malarial drug halofuginone and the antibiotic mupirocin.

These approaches have two other uses. One is that they can be used for validating chemical-genetic interactions. For compounds that have putative mechanisms of action, often based on *in vitro* biochemical assays, strains with depletions of possible targets or potential activators can be constructed using either targeted protein degradation or CRISPRi. This permits mapping to the true target in whole cells, as discussed below. Conversely, specifically constructed strains can aid in targeted drug discovery using a whole-cell approach. For example, Evans et al. used a complementary genetic approach to transcriptionally silence multiple genes in the panthothenate and coenzyme A biosynthesis pathways (Evans et al., 2016). These strains were then used with chemical screening to discover whole cell-active inhibitors.

## Synergy: Using existing drugs in new ways

Target-based discovery has already offered new and promising insights to help simplify TB therapy and reduce treatment duration. By studying the interplay of drugs and specific targets, we can design more deliberate regimens with a focus on synergistic interactions. Synergy refers to a combined effect of multiple drugs that is greater than the sum of their individual effects. Although TB combination therapy was developed for several important reasons, synergy was not front of mind when designing current regimens. How can we incorporate a synergy-focused framework in the design of new TB treatments?

One way to do this is by screening genetic libraries against existing antibiotics to find genes whose depletion hypersensitizes *Mtb* to existing drugs that are either already used or currently ineffective against TB. Both deletion and depletion mutants can phenocopy antibiotics targeting the mutated protein, allowing us to screen for synergistic “drugs” without yet having a molecule in hand. In a recent TnSeq screen, transposon mutant libraries were screened in the presence of different antibiotics with diverse mechanisms of action (Xu et al., 2017). Multiple genetic determinants of antibiotic susceptibility were involved in synthesis and maintenance of *Mtb*’s cell envelope. For instance, deletion of the gene *fecB*, which mediates cell wall integrity, conferred susceptibility to every antibiotic tested. Findings like this suggest that efforts directed toward increasing permeability of *Mtb* cells would expand the arsenal of antibiotics that could potentially be used to treat TB by exploiting synergy between cell wall inhibitors and intracellular antibiotics. Similarly, another TnSeq screen performed in mice treated with the first-line TB drugs rifampicin, isoniazid, ethambutol, and pyrazinamide found that the bottleneck for rifampicin efficacy is permeability to the drug, whereas isoniazid susceptibility is predominantly affected by replication rates (Bellerose et al., 2020). These findings were also recently corroborated by observations that growth on cholesterol—a host-relevant carbon source—decreased susceptibility to rifampicin by causing lipid composition changes on *Mtb*’s cell envelope: efforts targeting cell wall synthesis pathways would enhance killing by rifampicin during infection (Koh et al., 2022).

Another chemical-genetic screen was recently performed using CRISPRi libraries. Expression levels of most *Mtb* genes were titrated and bacterial fitness quantified in the presence of different antibiotics (Li et al., 2021). A putative aminoglycoside transporter was identified when decreased levels of Rv1819c (*bacA*, an ABC importer of hydrophilic solutes) conferred resistance to streptomycin, showing once again the importance of understanding determinants of drug resistance and the promise of increasing cell wall permeability to improving *Mtb* susceptibility to antibiotics.

In addition to chemical-genetic screens to identify mechanisms of synergy between putative drug targets and existing antibiotics, combinatorial drug screening offers an approach to quantify synergy and prioritize drug combinations to test *in vivo*. New experimental and analytical methodologies have strengthened our ability to measure drug interactions. Checkerboard assays have historically been the gold standard for measuring pairwise drug interactions. These assays test antibiotics in double serial dilutions and compare their combined effect with each drug’s individual effect in a microtiter plate. The recent development of the more efficient and less expensive DiaMOND (Diagonal Measurement Of N-way Drug interactions) offers a relatively simple way to measure interactions between any number of drugs (Cokol et al., 2017). This technique compares dose responses for mixtures of drugs with dose responses of each individual drug using a Loewe additivity model. DiaMOND’s efficiency comes from applying geometric models to factor high-order drug interactions into lower-order components, creating a framework to predict higher-order interactions. Synergy studies *in vitro* have been extended to prioritize experimental drug combinations *in vivo*. Recent work by Larkins-Ford et al. identified signatures of drug potency and interactions in *in vitro* models that were predictive of efficacy in preclinical mouse models of TB (Larkins-Ford et al., 2021). The ability to identify synergy and antagonism between different drugs *in vitro* will be extremely helpful in designing future combination drug regimens against TB, especially as more drug targets enter the discovery pipeline. Drugs that bolster each other in combination will likely be more effective in clearing infections and have fewer side effects in patients due to reducing both dosage and treatment time.

## Prioritizing candidate regimens

An obvious limitation to the design of new combination therapies is the financial and time cost of large-scale clinical trials to assess their efficacy. Thus, preclinical testing of TB therapies will need to address multiple important factors to prioritize regimens for clinical assessment: (1) *in vitro* efficacy on total killing in different bacterial subpopulations and growth conditions and (2) their efficacy in animal models of TB relapse.

A major challenge to TB treatment is the heterogeneity of bacterial populations within a host and host environments. As described above, TB therapy is administered for several months because *Mtb* creates phenotypically diverse subpopulations with varying levels of drug tolerance. This is a result both of *Mtb*’s metabolic adaptation to different microenvironments within a host such as different carbon sources and other changes between granulomas, as well as *Mtb*’s asymmetric growth and division pattern [reviewed in (Chung et al., 2022)]. For instance, genetic knockdown of *lamA*, which influences cell size variation in mycobacteria, increases *Mtb* susceptibility to rifampicin and vancomycin (Rego et al., 2017). Assays that measure total bacterial killing or the rate of bacterial killing—as opposed to minimal inhibitory concentrations, the concentration at which >90% of bacterial growth is inhibited in a broth microdilution assay—might be more informative about the bactericidal dynamics of small molecules. Recent progress with animal models has also increased our ability to assess applicability of *in vitro* findings to *in vivo* models. Infection relapse models, in which mice are monitored for culturable bacteria after stopping drug treatment, are an important way to test the total sterilizing activity of a potential therapy (Larkins-Ford et al., 2021). Work on understanding the metabolic changes that *Mtb* undergoes in different microenvironments demonstrates the importance of testing compounds in different growth conditions including various carbon sources, pH, and hypoxia (Hicks et al., 2018; Gouzy et al., 2021). Future work continuing to understand how these subpopulations form will facilitate the discovery of putative drug targets that synergize to fully sterilize all infected microenvironments.

## Conclusion

In this review, we have outlined the limitations of current multidrug regimens against tuberculosis in the era of antibiotic resistance and the genetic tools available to improve the identification of drug targets and assess their vulnerability in *Mtb*. The rational design of regimens that sterilize diverse phenotypic subpopulations will maximize bacterial killing while minimizing both treatment duration and infection relapse. Importantly, the TB field currently has all the necessary genetic and analytical tools to screen for and prioritize drug targets *in vitro* based on the vulnerability of essential and non-essential genes in the *Mtb* genome and to translate these findings in *in vivo* models. Combining genetic methods with chemical screens offers a formidable strategy to redefine the preclinical design of TB therapy by identifying powerful new targets altogether, as well as targets that lend new efficacy to existing drugs.

## Author contributions

Conceptualization and outline – FGT and EJR. Writing – Original Draft: FGT. Writing – Review and Editing: FGT, EJR. All authors contributed to the article and approved the submitted version.

## Funding

This work was supported in part by NIH/NIAID through award P01AI095208, and by the Office of the Assistant Secretary of Defense for Health Affairs, through the Peer Reviewed Medical Research Program, Focused Program Award under Award No. W81XWH-17-1-0692. Opinions, interpretations, conclusions and recommendations are those of the author and are not necessarily endorsed by the Department of Defense.

## Conflict of interest

The authors declare that the research was conducted in the absence of any commercial or financial relationships that could be construed as a potential conflict of interest.

## Publisher’s note

All claims expressed in this article are solely those of the authors and do not necessarily represent those of their affiliated organizations, or those of the publisher, the editors and the reviewers. Any product that may be evaluated in this article, or claim that may be made by its manufacturer, is not guaranteed or endorsed by the publisher.
